# Pre-stroke socioeconomic status predicts upper limb motor recovery after inpatient neurorehabilitation

**DOI:** 10.1080/07853890.2022.2059557

**Published:** 2022-05-05

**Authors:** S. Wolf, S. E. Holm, T. Ingwersen, C. Bartling, G. Bender, G. Birke, A. Meyer, A. Nolte, K. Ottes, O. Pade, M. Peller, J. Steinmetz, C. Gerloff, G. Thomalla

**Affiliations:** a Department of Neurology, University Medical Center Hamburg-Eppendorf, Hamburg, Germany; b Department of Neurology, Charité – University Medicine Berlin, Berlin, Germany; c Clinic for Neurological Rehabilitation, MEDICLIN Klinikum Soltau, Soltau, Germany; d Department Neurology, RehaCentrum Hamburg, Hamburg, Germany; e Department Neurology, VAMED Klinik Geesthacht, Geesthacht, Germany; f Clinic for Neurological Rehabilitation, Klinikum Bad Bramstedt, Bad Bramstedt, Germany; g Department Neurology, VAMED Rehaklinik Damp, Damp, Germany

**Keywords:** Stroke, socioeconomic status, neurological rehabilitation, recovery of function, upper extremity, patient reported outcome measures

## Abstract

**Background:**

Lower socioeconomic status (SES) is associated with higher mortality rates and the likelihood of receiving less evidence-based treatment after stroke. In contrast, little is known about the impact of SES on recovery after discharge from inpatient rehabilitation. The aim of this study was to investigate the influence of SES on long-term recovery after stroke.

**Patients and methods:**

In a prospective, observational, multicentre study, inpatients were recruited towards the end of rehabilitation. The 12-month follow-up focussed on upper limb motor recovery, measured by the Fugl-Meyer score. A clinically relevant improvement of ≥5.25 points was considered recovery. Patient-centric measures such as the Patient-reported Outcomes Measurement Information System-Physical Health (PROMIS-10 PH) provided secondary outcomes. Information on schooling, vocational training, income and occupational status pre-stroke entered a multidimensional SES index. Multivariate logistic regression models calculating odds ratios (ORs) and corresponding confidence intervals (CIs) were applied. SES was added to an initial model including age, sex and baseline neurological deficit. Additional exploratory analyses examined the association between SES and outpatient treatment.

**Results:**

One hundred and seventy-six patients were enrolled of whom 98 had SES and long-term recovery data. Model comparisons showed the SES-model superior to the initial model (Akaike information criterion (AIC): 123 vs. 120, Pseudo *R*^2^: 0.09 vs. 0.13). The likelihood of motor recovery (OR = 17.12, 95%CI = 1.31; 224.18) and PROMIS-10 PH improvement (OR = 20.76, 95%CI = 1.28; 337.11) were significantly increased with higher SES, along with more frequent use of outpatient therapy (*p* = .02).

**Conclusions:**

Higher pre-stroke SES is associated with better long-term recovery after discharge from rehabilitation. Understanding these factors can improve outpatient long-term stroke care and lead to better recovery.

**KEY MESSAGE**
- Higher pre-stroke socioeconomic status (SES) is associated with better long-term recovery after discharge from rehabilitation both in terms of motor function and self-reported health status.
- Higher SES is associated with significantly higher utilization of outpatient therapies.
- Discharge management of rehabilitation clinics should identify and address socioeconomic factors in order to detect individual needs and to improve outpatient recovery.
- **Article registration:** clinicaltrials.gov NCT04119479.

## Introduction

Social inequalities, particularly those related to socioeconomic status (SES), are emerging as important determinants of health and require increased scientific attention. To date, health inequalities within and between countries persist, although they could be reduced and their importance is increasingly well understood [1–3]. This is also true for countries with reasonably high health standards, such as Germany [4]. Thus, it is clear that addressing these inequalities requires policy development of health systems and, as a foundation, a broad knowledge base that identifies and examines the relevant determinants [1,5,6].

As stroke is a leading cause of death and disability worldwide, it is particularly important to understand factors contributing to persistent impairments in functional status and quality of life in this condition [7–9]. Lower SES is associated with higher burden of cardiovascular risk factors, as well as higher stroke incidence and mortality [10–12]. In addition, patients with lower SES have more severe deficits and are less likely to receive effective treatments according to current guidelines [7]. Nevertheless, in stroke studies, data on SES are still rarely collected, reported and evaluated in a structured way [13]. This especially applies to the period after inpatient stroke rehabilitation, where systematic data are scarce, anyway. In a previous study, inequalities in the rehabilitation phase of stroke care after discharge were determined by patients' income (IN) status [14]. Access to good rehabilitative care for stroke patients appeared to be, at least partially, predetermined by SES in another study [15].

There is evidence of an association between SES and overall functional disability after stroke, as assessed, for example, by the Barthel index or the modified Rankin score [4,16]. However, achieving clinically meaningful upper limb function, that is satisfactory to the patient, is of immense importance to long-term recovery from stroke. This is especially true for younger patients who still have a prospect of returning to work, which is also interlinked with SES [17]. Hence, in particular, hand-arm motor function is a key factor for the performance of activities of daily living (ADLs) and returning to work [18,19]. In this context, the patient-centric view of their own recovery should not be neglected alongside objective measures of upper limb function. Indeed, as a result, the focus of clinical research lies increasingly on patient-reported outcomes, such as the Patient-reported Outcomes Measurement Information System 10-Question Short Form (PROMIS-10) [20,21]. But still little is known about the interaction of SES and outcome after rehabilitation for stroke.

In order to address this question, we studied the effect of SES on long-term upper extremity recovery. For this purpose, we measured SES as a multidimensional construct based on educational attainment, occupational status and/or IN as suggested before [10,22]. The primary objective of the present study was to examine whether SES prior to stroke impacts the likelihood of upper extremity motor recovery in mildly affected patients. It is hypothesized that with higher SES, there is higher potential for better long-term recovery after stroke. Secondary outcomes examined were the dependencies between patient-centred, self-reported outcomes and SES. In additional explanatory analyses, we also aimed at evaluating the amount of outpatient therapy use in this context. We focussed specifically on the period after discharge from inpatient neurological rehabilitation, a period for which few longitudinal data are available.

## Patients and methods

### Setting and subjects

The current analysis was part of the IMPROVE project – Interdisciplinary Platform for Rehabilitation Research and Innovative Care of Stroke Patients [23]. The aim of this project was to observe the long-term dynamics of stroke recovery to better understand the relationships between clinical and demographic factors and functional outcomes. This observational, longitudinal, multicentre study was conducted at a university stroke centre in cooperation with five neurological rehabilitation centres in Germany and explored the post-inpatient phase of stroke rehabilitation. Patients who suffered from ischaemic or haemorrhagic stroke were enrolled between June 2017 and July 2019. The first examination was scheduled for the last two weeks of the inpatient stay at the rehabilitation centres. Follow-up examinations took place three, six and 12 months after inclusion at the university research facility.

Patients were included if they met the following criteria: (a) ischaemic or haemorrhagic stroke according to ICD 10 I61-I69, (b) patients in or after completion of rehabilitation phases C and D according to the criteria of the Bundesarbeitsgemeinschaft für Rehabilitation [24], (c) age ≥18, (d) sufficient knowledge of German language, (e) informed consent to participate in the IMPROVE study and (f) deficit still existing (modified Rankin score [25] of at least one at inclusion). Exclusion criteria were defined as follows: (a) need for care prior stroke (assessed by the study physician using a questionnaire: “was there a need for care prior to the stroke?”), (b) subarachnoid haemorrhage, cranio-cerebral trauma, transitory ischaemic attack as primary diagnosis, (c) severe pre-existing psychiatric disease and (d) participation in follow-up examination not possible.

Trained study personnel performed a comprehensive clinical assessment at all timepoints, containing scores commonly used in neurorehabilitation, like the Barthel index [26] or National Institutes of Health Stroke Scale (NIHSS), to quantify the overall impairment caused by stroke [27]. Data on stroke characteristics (e.g. type of stroke, lesion side or time after stroke), vascular risk factors (e.g. nicotine abuse, overweight, diabetes mellitus, hypertension, hyperlipidaemia or coronary artery disease) and demographic data (e.g. age and sex) were collected at enrolment. At the follow-up appointments, all participants were additionally asked by questionnaire about the outpatient therapy methods they used. Ambulatory therapy volume for physiotherapy and occupational therapy was assessed. Ambulatory therapy was considered to be therapy provided outside of specialized rehabilitation clinics, as it is provided in established practices (physiotherapy/occupational therapy). Patients were specifically asked about the frequency (per week) and the duration (minutes per week). For analysis, averages were calculated, combining physiotherapy and occupational therapy into a single variable: motor therapy.

If patients needed help in completing questionnaires, they were assisted by study staff. Otherwise, they were able to answer the questions independently and at their own leisure. The assessments and scores used reflect the three components of the International Classification of Functioning, Disability and Health (ICF). Thereby, the overall focus was on upper extremity recovery.

The study was carried out following the Helsinki Declaration of the World Medical Association and approval of the local ethics committees (ethical boards of the medical associations Hamburg, Schleswig-Holstein, Niedersachsen) was obtained (approval no. PV5483). The trial protocol was registered at ClinicalTrials.gov (NCT04119479). Informed written consent was obtained for the specific study reported here, which was conducted as part of the IMPROVE collaboration project.

### Assessment of recovery

#### Primary outcome

The Fugl-Meyer Upper Limb (FM-UL) score [28], which is most commonly applied in stroke rehabilitation [29], was used to assess upper limb motor recovery after stroke and served as the outcome of primary interest. The maximum total score is 66, indicating perfect recovery. In this study, the difference in FM-UL scores between the first visit (end of inpatient stay) and the last follow-up visit (one year after inclusion) was used to assess whether patients continued to recover. Depending on the individual change score, patients were assigned to one of two categories: recovery or non-recovery. The clinically important difference (CID) for overall upper limb function of ≥5.25 points was set as cut-off value to distinguish between non-recovery and recovery [30]. Since FM-UL only assigns integer scores and no decimal points, all change scores ≥6 are in fact considered as recovery.

#### Secondary outcomes

The Stroke Impact Scale (SIS) is a stroke-specific quality of life instrument that measures the consequences of stroke [31]. It is commonly applied as an outcome measure to determine the quality of life after stroke rehabilitation. SIS data were collected only at follow-up appointments, as most questions relate to patients' daily lives in the home environment. The hand function domain (SIS HF), reflecting the upper limb function self-perception, was analysed. As for FM-UL, the CID was used as cut-off for the assignment to recovery or non-recovery. All change scores equal to or above 17.8 points were defined as recovery [32].

The PROMIS-10, a patient-centred standard set to measure global health in stroke patients [33,34], was obtained via questionnaire at each visit. The change between baseline and one-year follow-up *T*-scores of the physical health domain (PROMIS-10 PH) was analysed. As no CID values are currently available for this assessment, the minimal detectable change (MDC) of ≥6.51 points was considered a meaningful change and served as cut-off value for non-recovery vs. recovery [35].

### Socioeconomic status

The inclusion questionnaire catalogue was screened for SES relevant questions. Only information describing the patient was used; information on the spouse or household was not included. Items representing the following four areas were extracted and merged into an SES index: school education (SE), vocational training (VT), IN and professional status (PS). Thereby, we assigned a scoring value to each category of the four domains (Table S3 in the supplementary). For the scoring and classification, we have adopted an established definition used by Lampert et al. [22]. Each of the four domains, SE, VT, IN and PS, was normalized using min–max approach. Afterwards they were averaged unweighted to a multidimensional sum score to give an overall index that can range from 0 (representing lowest possible SES) to 1 (representing highest possible SES). Score generation was limited to those who provided information in at least three of the four domains.

For the comparisons, e.g. of baseline characteristics, we decided to do a tripartite division into a low, middle and high socioeconomic group, as it is common when examining SES [e.g. 22]. To establish the thresholds, three groups were formed based on the distribution of SES values, with each group corresponding to one tercile.

### Statistics

Descriptive data and group comparisons were analysed using the compareGroups package [36]. Based on the type of data provided, it was decided which statistic to use: for continuous normal distributed variables (i.e. age), mean, standard deviation and *t*-test were calculated. For continuous non-normal distributed variables (i.e. Barthel’s index), the median, minimum and maximum and Kruskal–Wallis test were computed. Categorical values (i.e. lesion side), were analysed using the absolute and relative frequencies and Chi-squared test.

For primary outcome analysis, a first multivariate logistic regression model (model 1) was fitted with recovery as the dependent variable. Age, gender and baseline functional status (represented by the NIHSS) were included in the model as explaining variables. The selected variables, especially baseline impairment and age are considered essential for the prediction of stroke outcome [37,38]. Afterwards, a second model (model 2) was fitted, additionally including SES index as predictor variable.

The assumptions for logistic regression were manually tested with the following steps: (a) linearity assumption was evaluated by checking linear relationship between continuous predictor variables and the logit of the outcome, (b) screening for potential influential values was done by examining Cook’s distance and standardized residual error (absolute standardized residuals ≥3 were considered as outliers) and (c) correlation of predictors (multicollinearity) was assessed by variance inflation factors.

Estimation of goodness of fit was done using McFadden Pseudo *R*^2^ [39] and likelihood-ratio test [40]. To evaluate the statistical significance of each coefficient in the model, the Wald test was calculated by taking the ratio of the square of the regression coefficient to the square of the standard error of the coefficient [41].

The final model (model 2) was then also calculated using the secondary dependent variables (i.e. recovery or non-recovery, generated using PROMIS-10 PH or SIS HF change scores).

All analyses were performed in R version 4.0.3 [42] and R Studio [43]. Data visualization was done using the packages sjPlot [44] and MLeval [45].

## Results

Of 176 patients enrolled, SES could be calculated for 162. Of these, 41 were female, with a median of 52 days since the stroke. The majority were affected by ischaemic stroke, only 13 patients had suffered a haemorrhage. Left and right hemispheres were affected approximately equally. The average age was 58 years. SES group assignment gave the following distribution: 61 individuals with low, 54 with middle and 47 with high status. Except for the distribution of stroke types and the presence of the risk factor smoking, there were no significant differences in baseline characteristics (Table 1).

**Table 1.** Summary descriptive table of study participants, divided according to socioeconomic groups.

	All	Low	Middle	High	*p* overall
	*N* = 162	*N* = 61	*N* = 54	*N* = 47	
Age, mean (SD)	58.1 (10.1)	59.8 (8.80)	56.0 (10.1)	58.1 (11.3)	.14
Sex					.41
Female	41 (25.3%)	19 (31.1%)	12 (22.2%)	10 (21.3%)	
Male	121 (74.7%)	42 (68.9%)	42 (77.8%)	37 (78.7%)	
Time after stroke (days)	52.0 [24.0; 233]	51.0 [28.0; 233]	48.0 [29.0; 158]	57.0 [24.0; 146]	.30
Median [min; max]					
Type of stroke					.05
Haemorrhagic	13 (8.1%)	1 (1.7%)	7 (13.0%)	5 (10.6%)	
Ischaemic	148 (91.9%)	59 (98.3%)	47 (87.0%)	42 (89.4%)	
Lesion side					.18
Both	9 (5.6%)	5 (8.5%)	2 (3.7%)	2 (4.3%)	
Left	87 (54.4%)	25 (42.4%)	35 (64.8%)	27 (57.4%)	
Right	64 (40.0%)	29 (49.2%)	17 (31.5%)	18 (38.3%)	
Handedness					.73
Left	9 (6.0%)	2 (3.5%)	4 (8.0%)	3 (6.8%)	
Right	141 (93.4%)	54 (94.7%)	46 (92.0%)	41 (93.2%)	
Unknown	1 (0.7%)	1 (1.8%)	0 (0.0%)	0 (0.0%)	
Barthel index	100 [10.0; 100]	100 [40.0; 100]	100 [10.0; 100]	100 [45.0; 100]	.85
Median [min; max]					
NIHSS	2.00 [0.00; 12.0]	2.00 [0.00; 12.0]	2.00 [0.00; 9.00]	3.00 [0.00; 11.0]	.18
Median [min; max]					
Modified Rankin score					.62
1	85 (52.5%)	33 (54.1%)	30 (55.6%)	22 (46.8%)	
2	52 (32.1%)	17 (27.9%)	16 (29.6%)	19 (40.4%)	
3	23 (14.2%)	11 (18.0%)	7 (13.0%)	5 (10.6%)	
4	2 (1.2%)	0 (0.0%)	1 (1.9%)	1 (2.1%)	
FM-UL	58.5 [3.00; 66.0]	59.0 [4.00; 66.0]	58.0 [3.00; 66.0]	57.5 [4.00; 66.0]	.85
Median [min; max]					
Existing cardiovascular risk factors					
Nicotine	86 (53.4%)	39 (65.0%)	30 (55.6%)	17 (36.2%)	.01
Overweight	73 (45.3%)	31 (51.7%)	27 (50.0%)	15 (31.9%)	.09
Diabetes	30 (18.6%)	12 (20.0%)	6 (11.1%)	12 (25.5%)	.17
Arterial hypertension	130 (80.7%)	48 (80.0%)	44 (81.5%)	38 (80.9%)	.98
Hyperlipidaemia	104 (68.9%)	38 (67.9%)	39 (75.0%)	27 (62.8%)	.43
Family history of stroke	21 (13.2%)	10 (17.2%)	7 (13.0%)	4 (8.5%)	.42
Alcohol	16 (10.0%)	6 (10.0%)	5 (9.4%)	5 (10.6%)	1.00
Previous heart attack	4 (2.5%)	0 (0.0%)	3 (5.6%)	1 (2.1%)	.15
Previous stroke	13 (8.1%)	5 (8.3%)	6 (11.1%)	2 (4.3%)	.45
Previous TIA	6 (5.0%)	1 (2.0%)	2 (4.9%)	3 (10.0%)	.28
Atrial fibrillation	12 (10.0%)	6 (12.2%)	2 (4.9%)	4 (13.3%)	.38

FM-UL: Fugl-Meyer Upper Limb score; NIHSS: National Institutes of Health Stroke Scale; SD: standard deviation; TIA: transient ischaemic attack.

**Figure 1.**
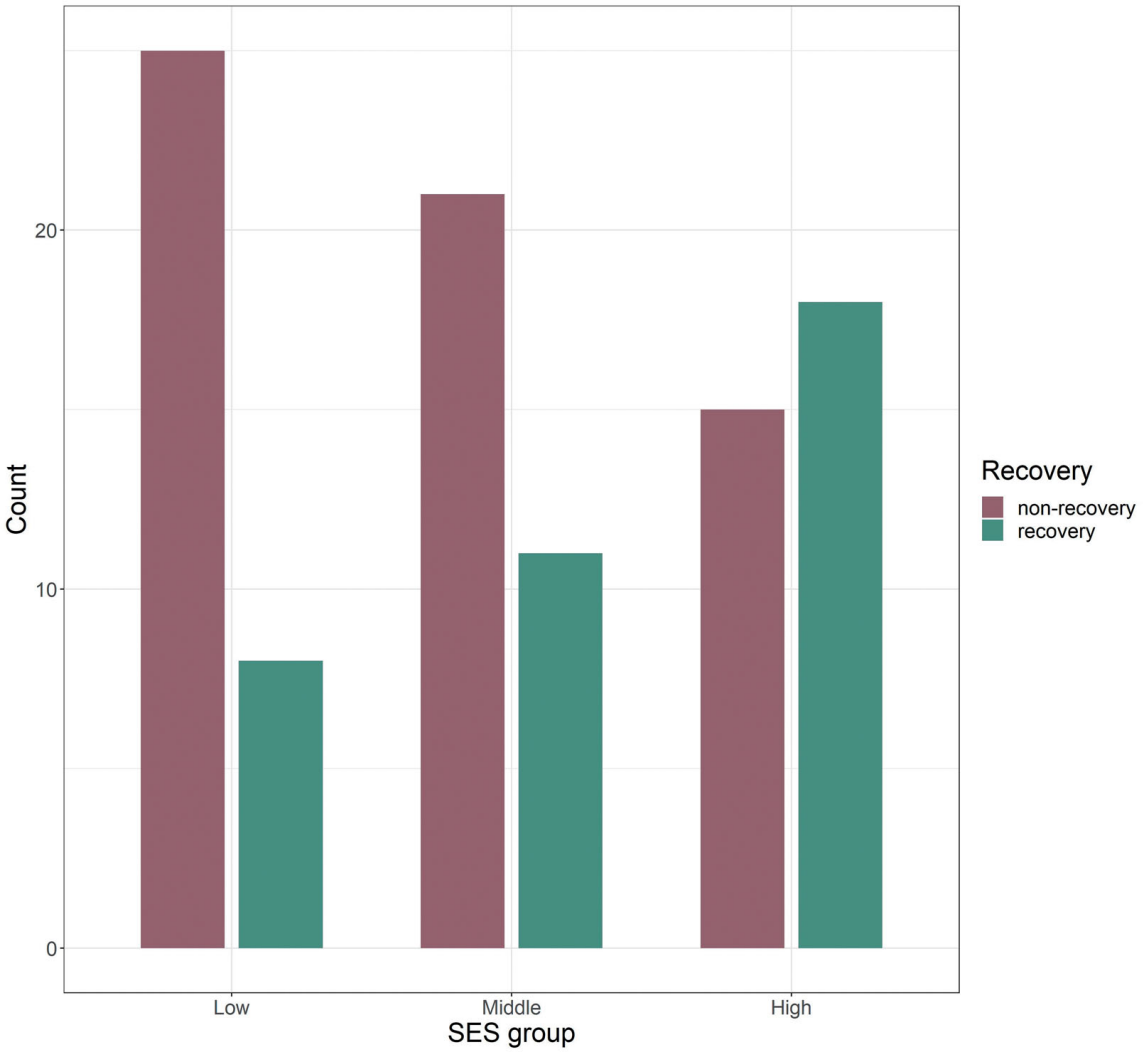
Distribution recovery and non-recovery within the three SES groups. SES: socioeconomic status.

**Figure 2.**
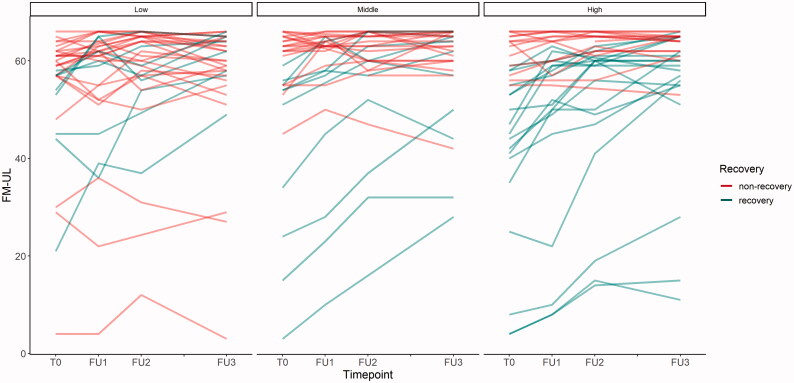
Progression of FM-UL scores over the observation period per SES group. FM-UL: Fugl-Meyer Upper Limb score; SES: socioeconomic status.

The group classification resulted in the following cut-off values for the SES index: 0.125–0.406 for low, 0.406–0.516 for middle and 0.516–0.917 for high status. Complete data on SES scores and one-year FM-UL change score were available for 98 patients (Figure 1). Thereof, 37 recovered, 61 did not recover or even got worse, as seen in the courses of FM-UL scores (Figure 2).

**Figure 3.**
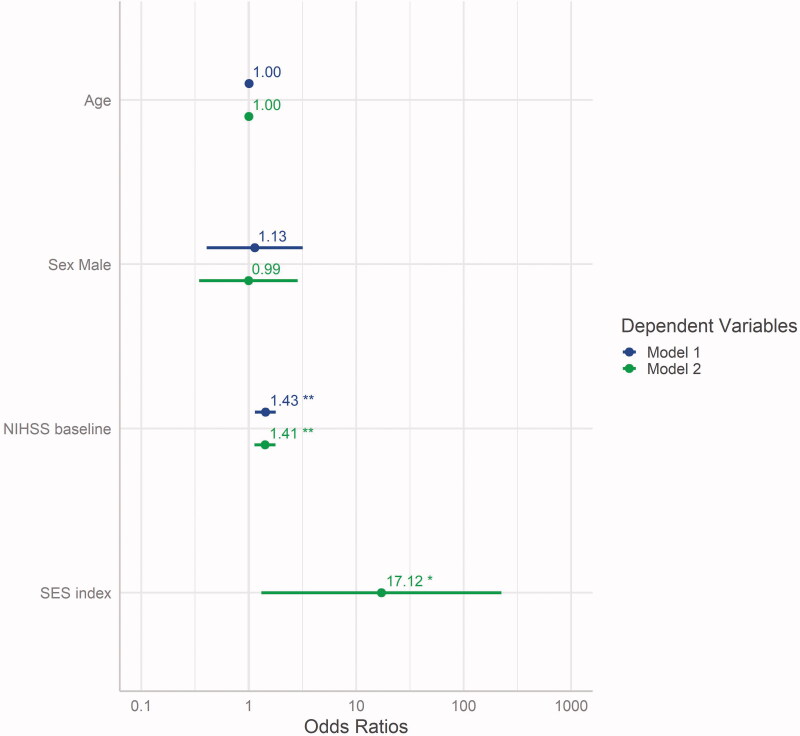
Estimates of model 1 compared to model 2 (additional predictor SES index), displayed with their respective 95% confidence intervals and significance level (**p < .01; *p < .05). NIHSS: National Institutes of Health Stroke Scale; SES: socioeconomic status.

As the prerequisite testing showed no abnormalities (Figures S2, S3 and S4 in the supplementary), the intended models could be calculated to predict recovery: model 1 (with age, sex and NIHSS at baseline) and the extended model 2 (including SES index as additional predictor). Model comparison showed that, in addition to the baseline stroke severity, SES had a significant predictive impact on the probability of further recovering from stroke (Figure 3, Table 2).

**Table 2.** Model comparison showing the exponentiated coefficients (i.e. adjusted OR) and 95% confidence intervals for predictors age, male sex, NIHSS at baseline and SES index.

	Model 1	Model 2
Age	1.00 (0.96; 1.05)	1.00 (0.96; 1.05)
Male sex	1.13 (0.41; 3.18)	0.99 (0.35; 2.86)
NIHSS	1.43** (1.14; 1.78)	1.41** (1.13; 1.77)
SES index		17.12* (1.31; 224.18)
*N*	96	96
AIC	123.19	120.27
Pseudo *R*^2^ (McFadden)	0.09	0.13

AIC: Akaike information criterion; NIHSS: National Institutes of Health Stroke Scale; OR: odds ratio; SES: socioeconomic status.

* *p* < .05.

** *p* < .01.

Goodness-of-fit testing, determined using the likelihood-ratio test, showed that model 2 was superior to the null model (*p* = .002) and to model 1 (*p* = .03) (Table S2 in the supplementary). The Wald test revealed significant relevance for the coefficients NIHSS (*F* = 9.0, on 1 and 91 DF, *p* = .003) and SES index (*F* = 4.7, on 1 and 91 DF, *p* = .033), but not for age (*F* = 0.002, on 1 and 91 DF, *p* = .964).

Fitting model 2 with the secondary endpoints as dependent variables showed that SES was significantly associated with the probability of recovery of PROMIS-10 PH, but not with recovery measured by SIS HF (Table 3).

**Table 3.** Final model 2 calculated for the secondary endpoints showing the exponentiated (i.e. adjusted OR) and 95% confidence intervals for predictors age, male sex, NIHSS at baseline and SES index.

	Model 2 DV: PROMIS-10 PH change	Model 2 DV: SIS HF change
Age	0.95 (0.91; 1.01)	1.04 (0.99; 1.11)
Male sex	0.94 (0.31; 2.87)	0.41 (0.13; 1.29)
NIHSS	0.99 (0.80; 1.23)	1.06 (0.86; 1.31)
SES index	20.76* (1.28; 337.11)	1.42 (0.06; 31.34)
*N*	104	91
AIC	111.61	92.76
Pseudo *R*^2^ (McFadden)	0.08	0.06

AIC: Akaike information criterion; DV: dependent variable; NIHSS: National Institutes of Health Stroke Scale; OR: odds ratio; PROMIS-10 PH: Patient-reported Outcomes Measurement Information System 10-Question Short Form-physical health domain; SES: socioeconomic status; SIS HF: Stroke Impact Scale-hand function domain.

* *p* < .05.

**Figure 4.**
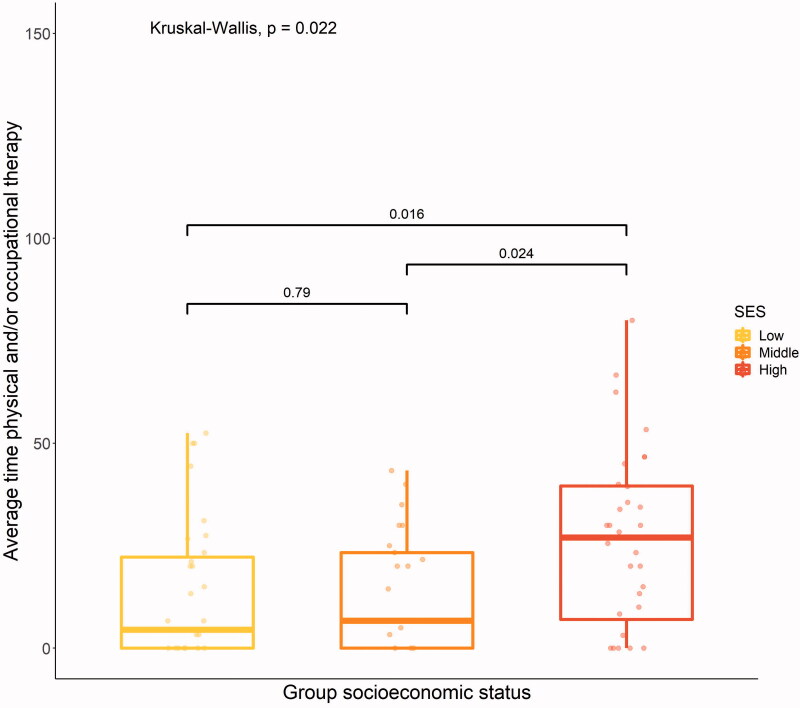
Average time per week (in minutes) for physical and/or occupational therapy, overall (Kruskal–Wallis) and between-groups (Wilcoxon) differences.

Individuals with higher SES used more physical and/or occupational therapy (*p* = .02) after discharge from rehabilitation than individuals from the other two groups (Figure 4).

## Discussion

In this analysis, we examined the impact of pre-stroke SES on long-term upper limb recovery after stroke rehabilitation. SES, comprising information on education, occupational status and IN, was a significant independent predictor for the likelihood of continued recovery in terms of motor function (measured by FM-UL) and self-reported physical health status (measured by PROMIS-10 PH) one year after completion of inpatient rehabilitation. Higher SES was further associated with more frequent use of outpatient therapies (such as physiotherapy and occupational therapy).

These findings fit with previous of an association between higher educational attainment and better motor and functional recovery during inpatient rehabilitation [14]. Our results indicate that the outpatient recovery course of stroke patients, especially mildly affected younger patients with the perspective of returning to work, in part depends on the pre-stroke SES. This is a novel finding and adds to the known association of lower SES with higher stroke incidence rates and more severe initial impairment [7].

In this paper, a significant and crucial emphasis is placed on the classification of motor recovery. Using the Fugl-Meyer value for this categorization must therefore be critically discussed. On the one hand, the instruments’ psychometric properties (such as the ordinal nature of the items or the existence of floor and ceiling effects) require more complex analysis methods. A high-resolution and specific measurement of movement quality is hardly possible. Therefore, there is an increasing demand for other outcome measurement tools in stroke research (such as kinematic motion analyses) [46]. On the other hand, the FM-UL remains widely established and is still very commonly used for outcome measurement in stroke rehabilitation [29]. We accordingly decided to use this instrument to assess motor recovery. A more conservative approach was chosen by defining a change value that was meaningful to the patient as a threshold for recovery. The consequence of this procedure is that some patients are technically unable to recover. In our cohort, which had already recovered very well at the time of inclusion, this concerned 14 individuals who already achieved 60 or more points at the time of inclusion and did not deteriorate over time to the one-year follow-up and did not achieve the full score. Excluding these 14 from the analysis, the association of higher SES with further recovery remained high, though the confidence intervals (CIs) now marginally included 1, likely due to the smaller number of cases (Figure S5 and Table S5 in the supplementary).

In contrast to the use of a cut-off, a purely numerical change in the score could also have been used. However, for the present analysis, a relevant measure was considered important that could also imply changes in patient management [47]. Different CID values for FM-UL can be found in the literature [e.g. 48,49]. Since our population is rather mildly affected, we opted for the value given by Page et al. [30]. Related to this, it is essential to consider those individuals in the sample who were more severely affected by stroke. At the time of inclusion, five patients had FM-UL scores below 15, but we nevertheless decided not to exclude these individuals from the analysis because all but one showed sustained long-term recovery (mean 15.5 points FM-UL). This was despite the fact that the stroke had occurred some time ago at the time of inclusion in the study (mean 116 days, range 51–146). These cases further illustrate the importance of long-term studies after stroke.

Indeed, the present work also aims to contribute to the expansion of knowledge on long-term outcome after stroke. Published literature mostly focuses on the acute and subacute phase after stroke, because this is when most recovery is expected and takes place and when patients are more easily within the reach of rehabilitation researchers [50]. Previous studies on long-term outcomes describe a deterioration of functional and motor outcomes months to years after stroke [51]. Thus, a stabilization of functional outcome in the long term might already be considered a success. This renders the continued recovery in a large proportion of our patients even interesting. These results reinforce that the critical period for recovery and potential interventions should not be restricted to the acute and subacute phase [52]. More investigations, such as those presented here, are needed to reduce uncertainties and also to focus more on the chronic course.

In further exploratory analysis, we found that in our population, the utilization of outpatient therapy services also was associated with SES, as is the case for access to acute care and rehabilitation [11,53,54]. Underutilization of support from the healthcare systems may provide a mechanism to explain the association between lower SES and worse outcomes. This is of specific interest, as it might provide a possible starting point to improve recovery from stroke. Identifying the needs of patients with lower SES may have an important impact on the delivery of patient-centred interventions in the chronic phase of stroke, as has been reported for post-acute recovery [16].

Previous work on SES and stroke suggests that disparities in post-stroke recovery may be due to differences in stroke severity and deficit at stroke onset. Patients with higher SES tended to have less severe strokes, as defined by NIHSS scores [55]. In our cohort, as in Grube et al. [4], no significant baseline difference was found between SES groups in stroke severity and therefore cannot explain the different recovery profiles. It should be noted, however, that the measured baseline severity in our cohort refers to the status at the end of inpatient rehabilitation which may limit the comparability of our results to those from previous studies.

The rather low proportion of women in our cohort (25%) also limits the generalizability of the results. The underrepresentation of women in clinical trials is a widely recognized bias in many different research areas. The problem of inadequate recruitment of women also exists in stroke studies, yet the causes of these disparities are complex and there remain relatively few stroke studies that have examined the causes in detail [56]. However, at least for severe strokes, there seems to be no difference in response to rehabilitation between women and men [57].

Another point of concern is the drop-out rate. Of the 162 patients included, complete analyses could only be performed for 96. Lost to follow-up and incomplete data sets are unfortunately always a problem when conducting longitudinal studies. However, a descriptive sensitivity analysis of our cohorts showed that there were no differences in the independent variables between the groups included vs. analysed (Table S4 in the supplementary).

To date, little is known about the interaction of self-reported health measures and SES in the context of stroke and neurorehabilitation [58]. A key explanatory factor for this is that little is known about stroke survivors' recovery after leaving inpatient rehabilitation in general. Data on this late stage of stroke recovery are still sparse, although this phase also holds much potential for patients and is worth understanding in more depth.

From a methodological point of view, the lack of an accepted universal operationalization of SES is a challenge and must be considered when comparing results between studies. Existing concepts are diverse, due to access to and use of a wide variety of data sources and a wide range of research questions [59]. While many studies consider only selected factors, such as education, occupation or IN, each of these factors represents an important component of SES [60]. For example, the measurement of total IN can provide good information about a person's current financial status, but education and occupation are poorly represented by this measure. Even though the latter are important influencing factors [15]. Considering IN as the only criterion may not be sufficient, also due to data collection problems like social desirability [16,61,62]. Established scores, the use of single indicators, or, like here, pragmatic approaches, each have advantages and disadvantages [61]. Here, for the purpose of conducting comparisons, we opted for a tripartite division into low, middle and high socioeconomic groups. This was done with the aim of forming three equally distributed groups. Other works, for example, use five-group division and then form a middle group from the three middle quintiles [22]. But the frequent use of group assignments and scoring systems, to allow for making generalized statements, is contrasted with the requirement to measure as much relevant socioeconomic information as possible and ideally to specify the individual socioeconomic factors measured [61]. Hence, there is a need for good and easy-to-use indexes, but there is also evidence that the use of index measures obscures the effects of the individual variables included in an index and thus prevents the detection of differential correlations [63]. In our analysis, we have tried to strike a balance that is both comprehensive and practical by integrating the available information on the different factors in a simple index.

## Conclusions

Higher SES was associated with a higher probability of motor function recovery and self-reported physical health status one year after stroke rehabilitation in our study. This could be related to more frequent utilization of outpatient treatments with higher SES. These findings highlight the need to identify patient-related factors that influence stroke such as SES in post-stroke care, as demonstrated in other work on this topic [4,7,14,54]. Since the focus of neurorehabilitation is primarily on interventions that serve to improve the physical and psychological impairments caused by stroke [64–66], this focus should expand towards more patient-centric approaches.

SES and social gradient appear to be relevant aspects for accessibility to treatment options, return to work after stroke and possibly also stroke recurrence [11,67,68]. Therefore, more attention should be paid to these factors both in research and clinical practice.

## Supplementary Material

Supplemental Material

## Data Availability

The raw data on which this article is based are intended for publication on an appropriate platform and can be made available by the authors upon reasonable request.
