# Supplementary material for: Pre-stroke socioeconomic status predicts upper limb motor recovery after inpatient neurorehabilitation
Source: Ann Med. 2022 May 5;54(1):1265–76. doi: 10.1080/07853890.2022.2059557 (PMC9090381; doi:10.1080/07853890.2022.2059557)
Supplement: Supplemental Material [file IANN_A_2059557_SM9444.docx]

# Supplement

## Distribution and progression of relevant variables

*
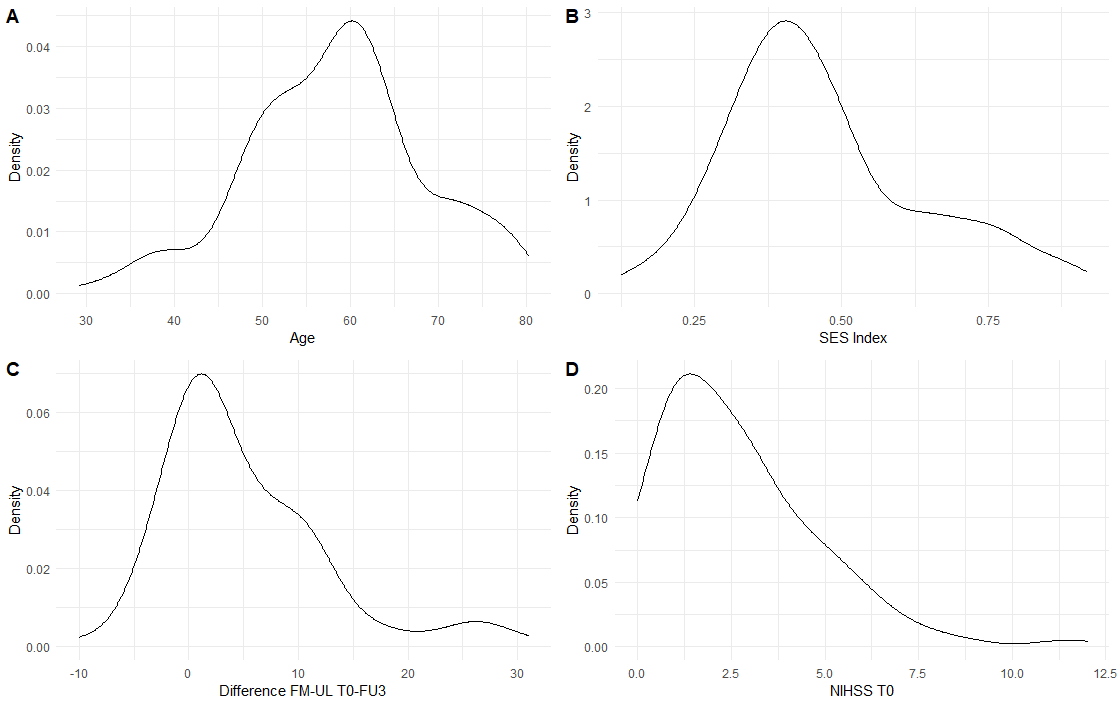
*

Figure S1: Density plots of A=Age, B=SES Index, C=Change Score Fugl-Meyer Baseline to one-year-follow-up, D=NIHSS baseline score

| SES Group  Recovery | Low | Middle | High | **Sum** |
| --- | --- | --- | --- | --- |
| Deteriorate  (FMA Difference Min - 0) | 8 (8.16%) | 5 (5.10%) | 5 (5.10%) | **18** |
| Stable  (FMA Difference 0 - 5) | 17 (17.35%) | 16 (16.33%) | 10 (10.21%) | **43** |
| Recovery  (FMA Difference 6 - Max) | 8 (8.16%) | 11 (11.23%) | 18 (18.37%) | **37** |
| **Sum** | **33** | **32** | **33** |  |

Table S1: Absolute values and percentages of patients who deteriorate, remained stable, and (continued to) recover in FM-UL change scores per SES group

## Assumptions for logistic regression


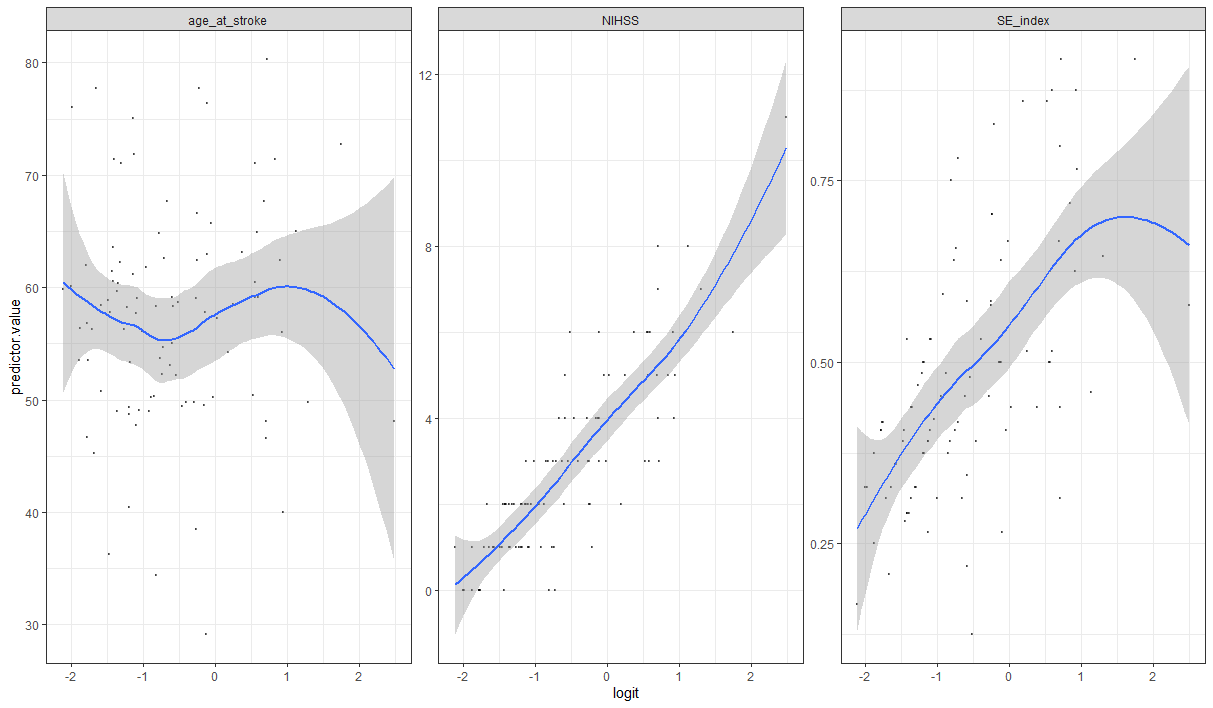


Figure S2: Checking of linearity assumption of continuous predictors with the logit of the outcome recovery

| 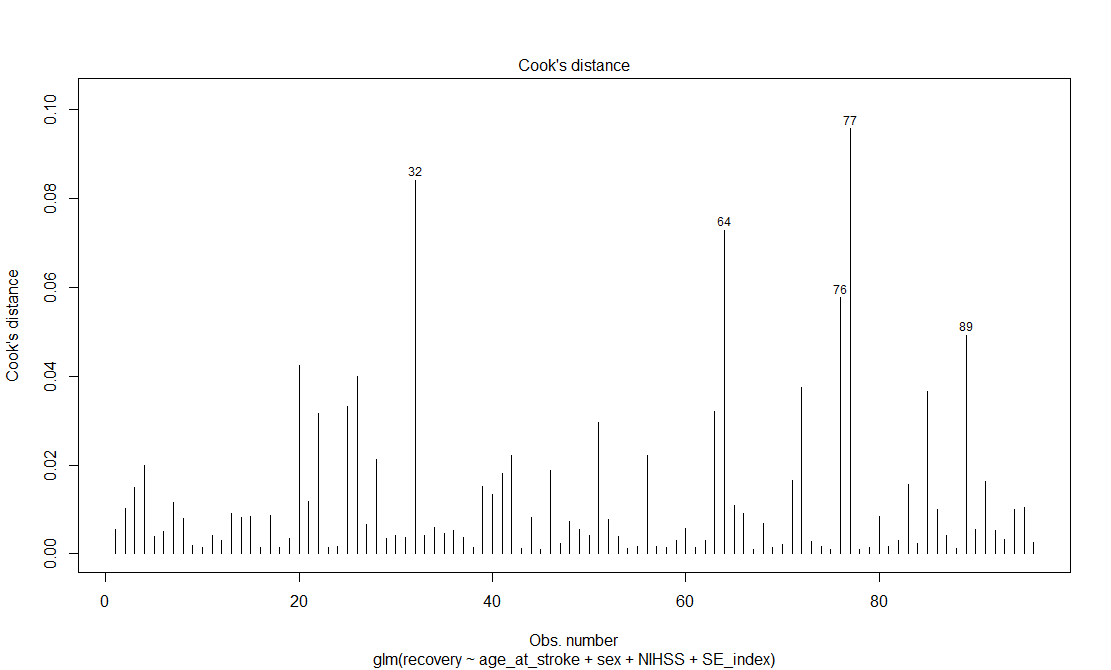 | 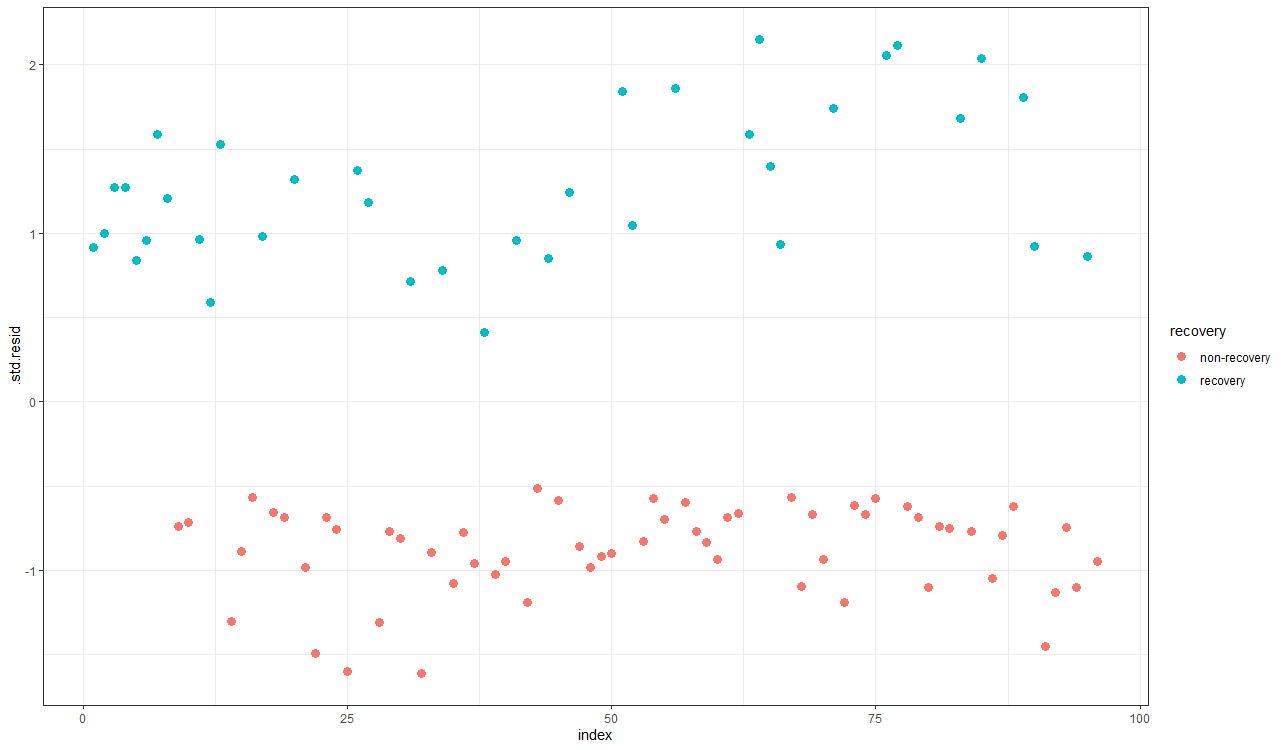 |
| --- | --- |

Figure S3: Checking of influential values, using Cook’s distance and standardized residuals


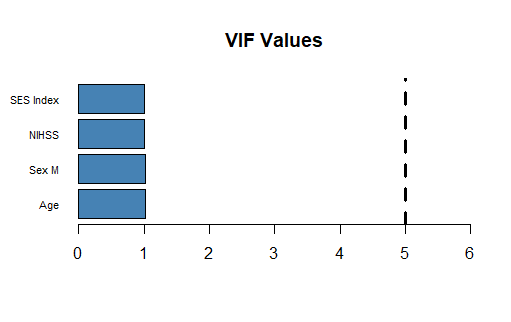


Figure S4: Checking for multicollinearity using variance inflation factors

## Goodness of fit – likelihood ratio test

| Likelihood Ratio Test: Model 2 against Null Modell |
| --- |
| Model 2: recovery ~ age_at_stroke + sex + NIHSS + SE_index  Model 0: recovery ~ 1  Df LogLik Df Chisq Pr(>Chisq)  1 5 -55.134  2 1 -63.510 -4 16.752 0.00216 ** |
| Likelihood Ratio Test: Model 2 against Model 1 |
| Model 1: recovery ~ age_at_stroke + sex + NIHSS  Model 2: recovery ~ age_at_stroke + sex + NIHSS + SE_index  Df LogLik Df Chisq Pr(>Chisq)  1 4 -57.596  2 5 -55.134 1 4.9246 0.02648 * |
| Significance codes: ‘**’ 0.01, ‘*’ 0.05 |

Table S2: Likelihood Ratio Test, Model 2 against Null Model and Model 1

## Questions and scoring for SES

| **Domain** | **school education (SE)** | **vocational training (VT)** | **professional status (PS)** | **Patient’s income (IN)** |
| --- | --- | --- | --- | --- |
| **Question** | What is your highest general education degree? | What vocational training qualifications do you have? | To which group does this occupation belong? | What is your monthly net income (€)? |
| **Categories and scoring** | "Left school without a secondary school leaving certificate (Volksschulabschluss)" = 1  "Hauptschulabschluss (elementary school diploma)" = 2  "Realschulabschluss (Mittlere Reife)" = 3  "Fachhochschulreife, completion of a specialized secondary school" = 4  "Allgemeine oder fachgebundene Hochschulreife/Abitur" = 5 | "No vocational qualification and I am not in vocational training" = 1  "Vocational training" = 2  "University degree or university of applied sciences degree (e.g. Diplom, Magister, Staatsexamen, Master, Bachelor)" = 3 | "Contributing family worker" = 1  "blue-collar worker" = 2  "Salaried employee" = 3  "Self-employed farmer or cooperative farmer" = 3  "Self-employed in trade, commerce, craft, industry, service" = 3  "Civil servant, judge, professional soldier" = 4  "Academic in independent profession (doctor, lawyer, tax consultant)" = 5 | "Under 500" = 1  "500 to under 750" = 2  "750 to under 1000" = 3  "1000 to under 1250" = 4  "1250 to under 1500" = 5  "1500 to under 1750" = 6  "1750 to under 2000" = 7  "2000 to under 2250" = 8  "2250 to under 2500" = 9  "2500 to under 3000" = 10  "3000 to under 3500" = 11  "3500 to under 4000" = 12  "4000 to under 4500" = 13  "4500 to under 5000" = 14  "5000 to under 6000" = 15  "6000 to under 8000" = 16  "8000 or higher" = 17 |

Table S3: Questions and response options with associated scoring for SES index

|  | **N=162 (T0)** | **N=96 (FU3)** |
| --- | --- | --- |
| Age – Mean (SD) | 58.1 (10.1) | 57.5 (9.7) |
| Sex  Female  Male | 41 (25.3%)  121 (74.7%) | 24 (25.0%)  72 (75.0%) |
| NIHSS – Median [Min;Max] | 2 [0;12] | 2 [0;11] |
| SES index – Mean (SD) | 0.47 (0.17) | 0.48 (0.18) |
| Additionally: SES group allocation | | |
| SES group  Low  Middle  High | 61 (37.7%)  54 (33.3%)  47 (29.0%) | 33 (34.4%)  30 (31.2%)  33 (34.4%) |

Table S4: Descriptive sensitivity analysis, included vs. analyzed subjects


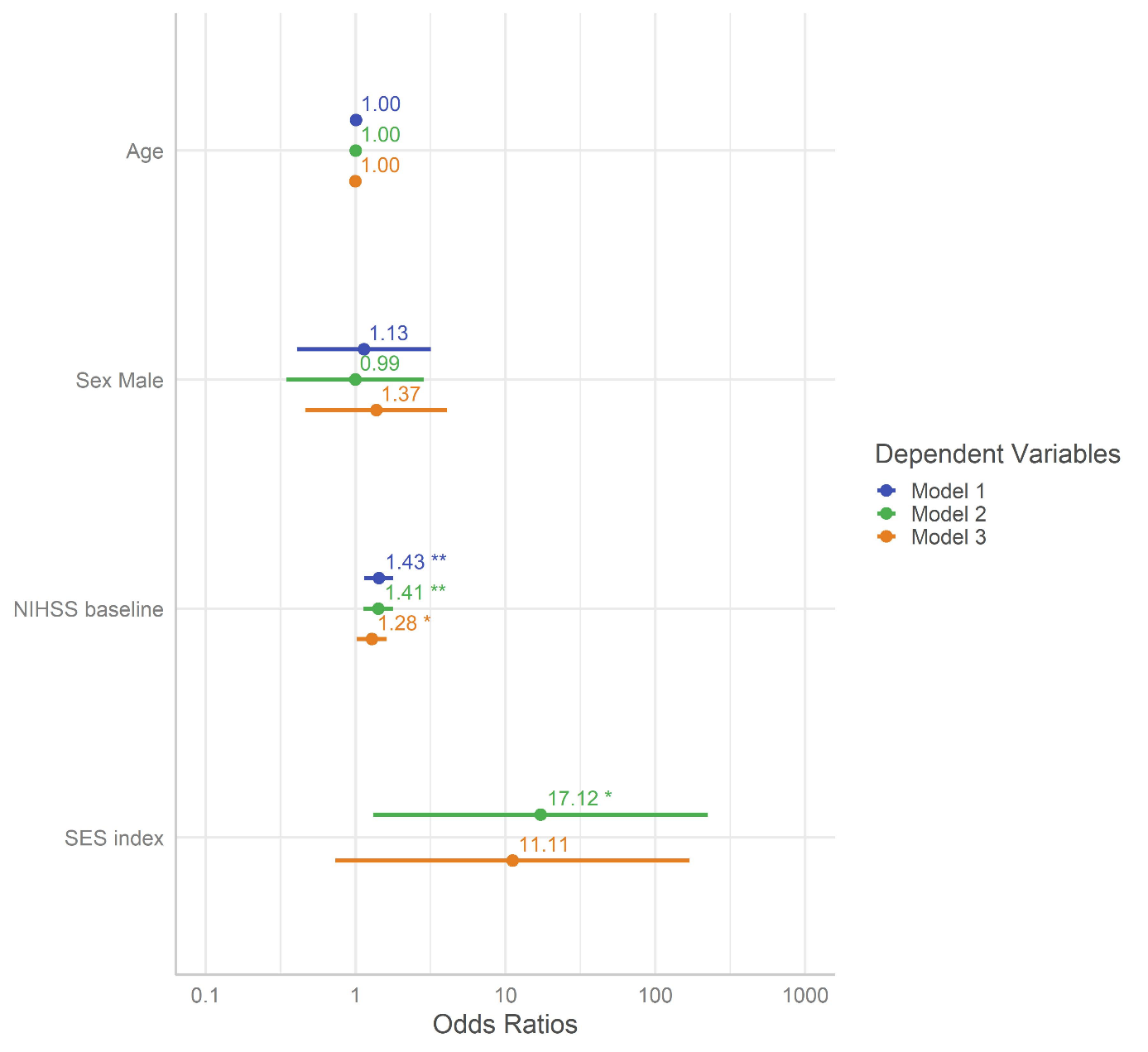


Figure S5: Calculating additional model 3 using a differnet sample: patients who could not meet the threshold for recovery (because they already had 60 or more points for inclusion) and did not achieve the full score were excluded

| **Age**  **Male sex**  **NIHSS**  **SES index**  **N**  **AIC**  **BIC**  **Pseudo R2** | **Model 2**  **───────**  **1.00**  **(0.96;1.05)**  **0.99**  **(0.35;2.86)**  **1.41 ****  **(1.13;1,77)**  **17.12 ***  **(1.31;224.18)**  **───────**  **96**  **120.27**  **133.09**  **0.13**  **───────** | **Model 3  (remove N=14 and assign N= 6**  **as recovery)**  **───────**  **1.00**  **(0.95;1.05)**  **1.37**  **(0.46;4.08)**  **1.28 ***  **(1.02;1.61)**  **11.11**  **(0.73;168.57)**  **───────**  **76**  **105.49**  **117.14**  **0.09**  **───────** |
| --- | --- | --- |
| **** p < 0.01; * p < 0.05, 95% CI in brackets** | | |

Table S5: Results of additionally calculated model 3 in comparison to model 2, for model 3 patients who could not meet the threshold for recovery (because they already had 60 or more points for inclusion) and did not achieve the full score were excluded
